# Optimising intraperitoneal gentamicin dosing in peritoneal dialysis patients with peritonitis (GIPD) study

**DOI:** 10.1186/1471-2369-10-42

**Published:** 2009-12-16

**Authors:** Dwarakanathan Ranganathan, Julie M Varghese, Robert G Fassett, Jeffrey Lipman, Vincent D'Intini, Helen Healy, Jason A Roberts

**Affiliations:** 1 Renal Medicine, Royal Brisbane and Women's Hospital, Brisbane, Queensland, 4029 Australia; 2 Burns, Trauma and Critical Care Research Centre, The University of Queensland, Royal Brisbane and Women's Hospital, Brisbane, Queensland, 4029 Australia; 3 School of Medicine, The University of Queensland, Brisbane, Queensland, 4027 Australia; 4 Pharmacy Department, Royal Brisbane and Women's Hospital, Brisbane, Queensland, 4029, Australia

## Abstract

**Background:**

Antibiotics are preferentially delivered via the peritoneal route to treat peritonitis, a major complication of peritoneal dialysis (PD), so that maximal concentrations are delivered at the site of infection. However, drugs administered intraperitoneally can be absorbed into the systemic circulation. Drugs excreted by the kidneys accumulate in PD patients, increasing the risk of toxicity. The aim of this study is to examine a model of gentamicin pharmacokinetics and to develop an intraperitoneal drug dosing regime that maximises bacterial killing and minimises toxicity.

**Methods/Design:**

This is an observational pharmacokinetic study of consecutive PD patients presenting to the Royal Brisbane and Women's Hospital with PD peritonitis and who meet the inclusion criteria. Participants will be allocated to either group 1, if anuric as defined by urine output less than 100 ml/day, or group 2: if non-anuric, as defined by urine output more than 100 ml/day. Recruitment will be limited to 15 participants in each group. Gentamicin dosing will be based on the present Royal Brisbane & Women's Hospital guidelines, which reflect the current International Society for Peritoneal Dialysis Peritonitis Treatment Recommendations. The primary endpoint is to describe the pharmacokinetics of gentamicin administered intraperitoneally in PD patients with peritonitis based on serial blood and dialysate drug levels.

**Discussion:**

The study will develop improved dosing recommendations for intraperitoneally administered gentamicin in PD patients with peritonitis. This will guide clinicians and pharmacists in selecting the most appropriate dosing regime of intraperitoneal gentamicin to treat peritonitis.

**Trial Registration:**

**ACTRN**12609000446268

## Background

Peritonitis is a major complication of PD and is one of the main reasons why patients are transferred to haemodialysis [1,2]. Antibiotics used to treat peritonitis may be delivered via the intraperitoneal (IP), intravenous (IV) or oral routes. In most cases of PD related peritonitis, the infection is localised to the peritoneum and cells lining the peritoneal cavity. The IP route of antibiotic administration is preferred because it ensures antibiotic concentrations at the local site/s of the infection are maximised [3]. However, drugs administered intraperitoneally may still be absorbed into the systemic circulation potentially leading to toxicity [4,5]. Peritonitis may also alter the permeability of the peritoneal membrane with an inflamed peritoneal membrane allowing increased absorption of drugs from the peritoneal cavity into the blood stream [6,7].

Most studies in this area have concentrated on the effectiveness and outcomes of IP antibiotic therapy in PD related peritonitis [8-13]. Only a few small studies have reported the pharmacokinetics of antibiotics delivered via the IP route [4,5,14-18]. Most were conducted in volunteer PD patients without peritonitis [4,5,16,18]. The absorption and clearance of drugs administered intraperitoneally are not well described, particularly in pathological states such as inflammation and infection. Poor or absent renal excretion adds complexity to the pharmacodynamics when drugs that reach the systemic circulation and are excreted by the kidneys, such as gentamicin, are used.

Gentamicin belongs to the aminoglycoside class of antibiotics and provides excellent coverage against Gram-negative bacteria. However, high blood levels are associated with nephrotoxicity and ototoxicity. In the past, gentamicin was not recommended for use in PD patients with residual renal function due to the risk of causing it to further decline [19]. A recent study has shown that the short term use of aminoglycosides is safe and does not compromise residual renal function [20]. This is reflected in the current International Society for Peritoneal Dialysis (ISPD) Guidelines/Recommendation for Peritoneal Dialysis-Related Infections published in 2005 where gentamicin is listed as one of the drug options for gram negative empirical antibiotic cover [3]. The recommended dose for intraperitoneal gentamicin in anuric patients is 0.6 mg/kg in a single dialysis bag administered once daily and allowed to dwell for at least six-hours [3].

Aminoglycosides display concentration dependent bacterial killing meaning maximal bacterial killing requires high peak drug concentrations [21] and also have a prolonged post-antibiotic effect (PAE) where bacterial growth continues to be inhibited even when the drug concentrations fall below the minimum inhibitory concentration [22]. Both the concentration dependent killing and the PAE justify the recommendations for once-daily gentamicin dosing [16,17]. IP antibiotic dosing according to current guidelines gives rise to high gentamicin concentrations in the peritoneal cavity for at least six-hours, which increases the likelihood of systemic absorption and subsequent toxicity without additional antibacterial effect.

A gentamicin trough concentration of <2 mg/L is usually recommended because early studies reported an increased risk of toxicity with higher concentrations [23]. A recent retrospective study from the UK found that the current ISPD dosing guidelines for IP gentamicin resulted in high trough plasma concentrations in over 50% of patients [9]. High plasma concentrations of gentamicin can not be rapidly removed by renal excretion in patients requiring PD increasing the risk of toxicity. Monitoring of gentamicin plasma concentrations in patients with renal impairment is useful in avoiding drug accumulation and minimising the possible toxicities of the drug, including loss of residual renal function.

This pharmacokinetic study of gentamicin was designed to develop improved dosing guidelines to achieve optimal bacterial kill, based on the performance of drug delivered intraperitoneally to a population with poor or no renal function.

### Aims

To describe the pharmacokinetics of gentamicin administered intraperitoneally in PD patients with peritonitis using a population pharmacokinetic model; and To test this pharmacokinetic model by computerised dosing simulations and subsequently develop dosing recommendations for IP gentamicin to optimise antibiotic efficacy and minimise toxicity.

## Methods/Design

### Study Design

This is a pharmacokinetic study. Samples of blood, dialysate fluid and urine will be collected from participants presenting to the Royal Brisbane and Women's Hospital (RBWH), diagnosed with PD peritonitis and treated according to protocol, based on the current International Society for Peritoneal Dialysis Peritonitis Treatment Recommendations.

### Setting

The Department of Renal Medicine, RBWH services a population of approximately 1.2 million. This study is done in collaboration with the Burns, Trauma and Critical Care Research Centre, School of Medicine, The University of Queensland.

### Identification of Eligible Patients

Participants who present with clinically defined signs and symptoms of peritonitis, as listed in Table 1, that fulfil the inclusion and exclusion criteria will be eligible for the study. Written informed consent will be obtained from each participant.

**Table 1:** Explanation of definitions to be used as part of this study

Terms	Definitions
**Peritonitis**	* Presence of two clinical signs and symptoms:
	abdominal pain, nausea, vomiting, diarrhoea, fever and cloudy dialysate
	* Peritoneal dialysate WCC > 100/mm3 with 50% neutrophils
	* Demonstration of bacteria on gram stain or culture

**Clinical failure**	* Insufficient lessening of signs and symptoms of infection to qualify as improvement
	* Continued symptoms or signs beyond day four
	* Dialysate WCC > 100/mm3 at day 14
	* Removal of the catheter for failure to respond to treatment
	* Recurrence of peritonitis with same micro-organism (relapse) within 28-day follow up period after cessation of antibiotics
	* Death due to uncontrolled infection

**Clinical success**	**Primary response **- disappearance of the signs and symptoms of peritonitis and clear, sterile PD on day 10
	
	**Relapsed **- primary response but recurrence by day 28
	
	**Complete cure **- no relapse by day 28 after completion of antibiotics

**Outcome indeterminate**	When no evaluation is possible for any reason

**Bacteriologic response based on culture results**	**Eradication **-Causative organisms absent and remaining absent for 28 days after completion of antibiotics
	
	**Persistence **- Causative organisms present at any culture dates after initiation of therapy
	
	**Superinfection **- Presence of new infecting organisms and cultures dates during and just after two days of therapy
	
	**Bacteriologic indeterminate **- When result not available for any reason including no growth in the first culture
	
	**Eradication with relapse **- Causative organisms absent at day 14 but present at or before 28 +/- 2 days follow up
	
	**Eradication with reinfection **- Causative organisms absent at day 14 and presence of new organisms at or before 28 +/- 2 days of follow up

Key: This is a list of suggested definitions for use in future research studies in this area- from the Caring for Australasians with Renal Impairment (CARI) Guidelines for PD associated peritonitis 2004

### Inclusion criteria

Male and female participants over 18 years of age, who have been receiving Continuous Ambulatory Peritoneal Dialysis (CAPD) or Automated Peritoneal Dialysis (APD) for more than one-month, presenting to the Royal Brisbane and Women's Hospital (RBWH) Renal Medicine Department with clinical evidence of peritonitis and requiring treatment with IP gentamicin according to the current RBWH protocol.

### Exclusion criteria

Participants will be excluded if their peritonitis does not require IP gentamicin based on the peritonitis protocol, are pregnant, planning a pregnancy or if they have received a course of antibiotics in the preceding two weeks.

### Participants

Participants will be stratified into two groups of 15 each according to residual urine output.

1. *Anuric participants *- with a urine output of less than 100 ml/day

2. *Non-anuric participants *- with a urine output of more than 100 ml/day

This will be based on the most recent 24-hour urinary collection done within the preceding six-month period. A collaborating 24-hour urinary collection will be performed, starting at the time of admission, to guide future gentamicin dosing.

### Drug dosing

Drug dosing will be based on the current International Society for Peritoneal Dialysis Guidelines/Recommendations for Peritoneal Dialysis-Related Infections (2005). The dose administered will depend on whether the participant is anuric or non-anuric.

#### Anuric participants

0.6 mg/kg gentamicin in a single two-litre PD bag administered intraperitoneally once daily and allowed to dwell for at least six-hours

#### Non-anuric participants

For this group of patients with residual renal function, the dose of gentamicin will be increased by 25% - 0.75 mg/kg for at least a six-hour dwell.

### Sample collection

All participants will have blood sampling on two separate days (day one and another day between day two and five of treatment). A cannula will be placed for serial plasma sampling and PD fluid will be collected as follows:

#### Sample collection on day one of treatment

Plasma samples (5 ml each) taken at one hour, three hours, six hours, seven hours and 24 hours after the start of draining in the first dialysate bag containing gentamicin.

PD fluid samples (5 ml) will also be collected at three hours and at the end of the dwell time at six hours.

#### Sample collection on one day between day two and five of treatment

Five millilitres of blood will be taken just prior to draining in the dialysate with gentamicin to measure the true 'trough' gentamicin levels. A further 5 mls of blood will be collected at one hour, three hours, six hours, seven hours and 24 hours after the administration of the bag with IP gentamicin. Five millilitres of PD fluid will also be collected prior to administration of next bag of gentamicin, at three hours and at the end of the dwell time. For each dose of IP gentamicin administered, the type and volume of dialysate and inflow rate of the dialysate loaded with drug will be recorded. A 5 ml sample from the preceding 24-hour urine collection will be collected to measure the concentration of gentamicin excreted in urine. Ten millilitre samples of PD fluid from each drained bag will also be obtained before the dialysis effluent is discarded.

### Sample handling and storage

Blood samples will be placed on ice and centrifuged at 3000 rpm before being stored at -80°C until analysis. Dialysate and urine samples will be stored at -80°C until analysis.

### Sample analysis

The Burns Trauma and Critical Care Research Centre of The University of Queensland will measure gentamicin levels in plasma and dialysate using validated liquid chromatography tandem-mass spectrometry (LC-MS/MS) analytical assays.

### Data collection

Additional data will be obtained by history from the participant or extracted from the clinical notes. These include:

1. Participant information - age, gender, weight, height, allergies, cause of kidney disease, co-morbidities

2. Microbiology results - Gram stain, culture and antimicrobial sensitivities of dialysate fluid at admission to hospital

3. Laboratory investigations - full blood count, electrolytes, coagulation profile, liver function test and C-reactive protein at admission to hospital as part of usual care.

4. Date of start of this period of peritoneal dialysis

5. Previous dialysis history

6. Other antibiotics prescribed concomitantly

7. Other drugs prescribed concomitantly

8. Details of gentamicin therapy, including dosing regime and duration

9. Length of stay in hospital

10. Outcome of treatment (clinical and/or bacteriological success) will be described using the definitions listed in Table 1

11. Renal function assessments -urine output measurement, 24-hour urinary creatinine clearance

### Statistical considerations

A sample size calculation including a power calculation is not required as this study is a non-interventional study and is not intended to compare between different treatments or interventions. The purpose of the study is to identify significant covariates that describe the variability in gentamicin pharmacokinetics in peritoneal dialysis participants. A sample size of 30 is sufficient for this population pharmacokinetic study to identify most clinically significant covariates. In general, population pharmacokinetic studies have fewer statistical design restrictions [24].

### Data analysis

The results of sample analysis will be entered into the pharmacokinetic computer software program NONMEM (GloboMax LLC, Hanover, MD, USA) to develop a model for gentamicin dosing via the intraperitoneal route. The influence of demographic and clinical covariates, including anuria, will be tested in the model. The model will simulate gentamicin pharmacokinetics for different dosing schedules to predict the best dosing recommendations for intraperitoneal gentamicin in peritoneal dialysis participants with peritonitis [25].

### Ethical Considerations

The Human Research and Ethics Committee of the Royal Brisbane and Women's Hospital (HREC/08/QRBW/8) and the Medical Research Ethics Committee of the University of Queensland (2009000673) have approved this study.

### Withdrawal from Study

Participants may withdraw from the study anytime without prejudice, as documented and explained at the time of consenting.

## Discussion

Multiple factors influence the absorption and clearance of IP antibiotics in peritoneal dialysis patients with peritonitis [24]. These include individual patient related variables like membrane transport characteristics and residual renal function, drug related factors such as the physicochemical properties of the drug itself and factors relating to the dialysis modality such as dialysate dwell time and the number of exchanges. The pharmacokinetic model that will be developed from this study will incorporate these factors and the information will be used to conduct dosing simulations of different antibiotic doses with different dwell times to determine the most effective dosing regimen for gentamicin delivered intraperitoneally. Optimised IP gentamicin dosing can shift the balance of our therapeutic interventions to maintain good clinical outcomes and lesser toxicity in this high risk patient population.

## Abbreviations

IP: Intraperitoneal; ISPD: International Society for Peritoneal Dialysis; MIC: Minimum Inhibitory Concentration; NONMEM: Non-Linear Mixed Effect Modelling; PAE: Post Antibiotic Effect; PD: Peritoneal dialysis.

## Competing interests

The authors declare that they have no competing interests.

## Authors' contributions

DR, JR and JV designed the study and wrote the protocol. JL and RGF provided advice and input and will be involved with the study. VD and HH provided advice and input and will be involved with the recruitment component of the study. All authors read and approved the final manuscript.

## Pre-publication history

The pre-publication history for this paper can be accessed here:

http://www.biomedcentral.com/1471-2369/10/42/prepub
